# Supplementary figures and images for: Repurposing of the enhancer-promoter communication underlies the compensation of Mesp2 by Mesp1
Source: PLoS Genet. 2022 Jan 13;18(1):e1010000. doi: 10.1371/journal.pgen.1010000 (PMC8791502; doi:10.1371/journal.pgen.1010000)

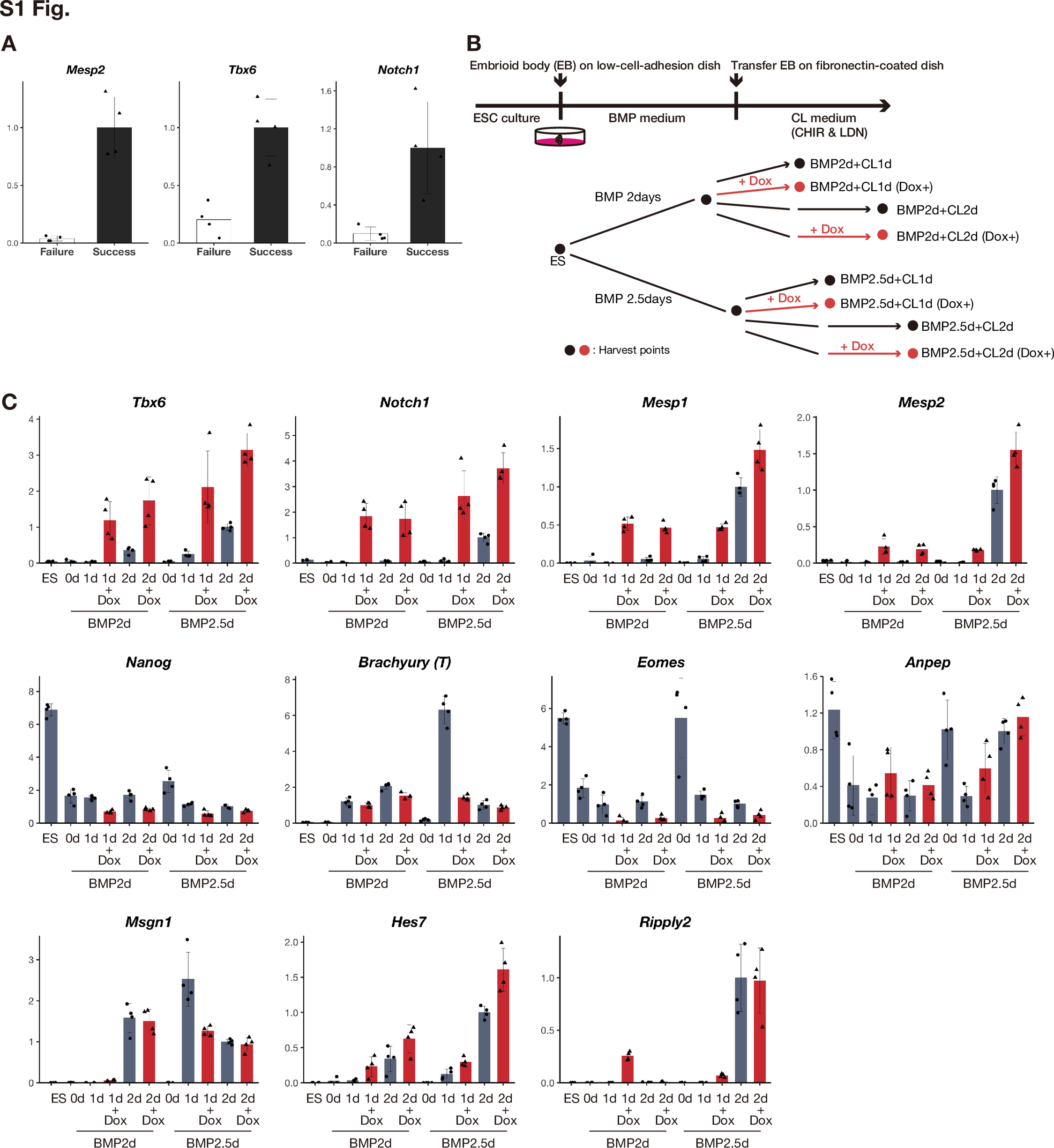

Supplement: S1 Fig — A qPCR analysis of Mesp2, Tbx6, and Notch1 in in vitro PSM using wild-type (WT) ES cells. Failure and Success indicate failed and successful PSM induction using the same WT ES cells (n = 4 cultures for each sample). The expression levels are presented as the ratio against the in vitro PSM successfully induced. B Schedule of in vitro PSM induction from ES cells to cell harvest to analysis. Dox administration is indicated in red. The duration of Dox administration was 24 hours in every set. Red and black circles are harvest time points with or without Dox administration, respectively. C qPCR analysis of representative developmental genes in iTbx6;iNICD during PSM induction with (red bars) or without (grey bars) Dox administration (n = 4 cultures for each sample). The expression levels are presented as the ratio against the in vitro PSM cultured in BMP for two and half days and CL media for two days without Dox administration. Nanog is a pluripotency marker; Brachyury (T) and Eomes are early mesoderm markers; Msgn1 and Hes7 are PSM markers. (TIF) [file pgen.1010000.s001.tif]

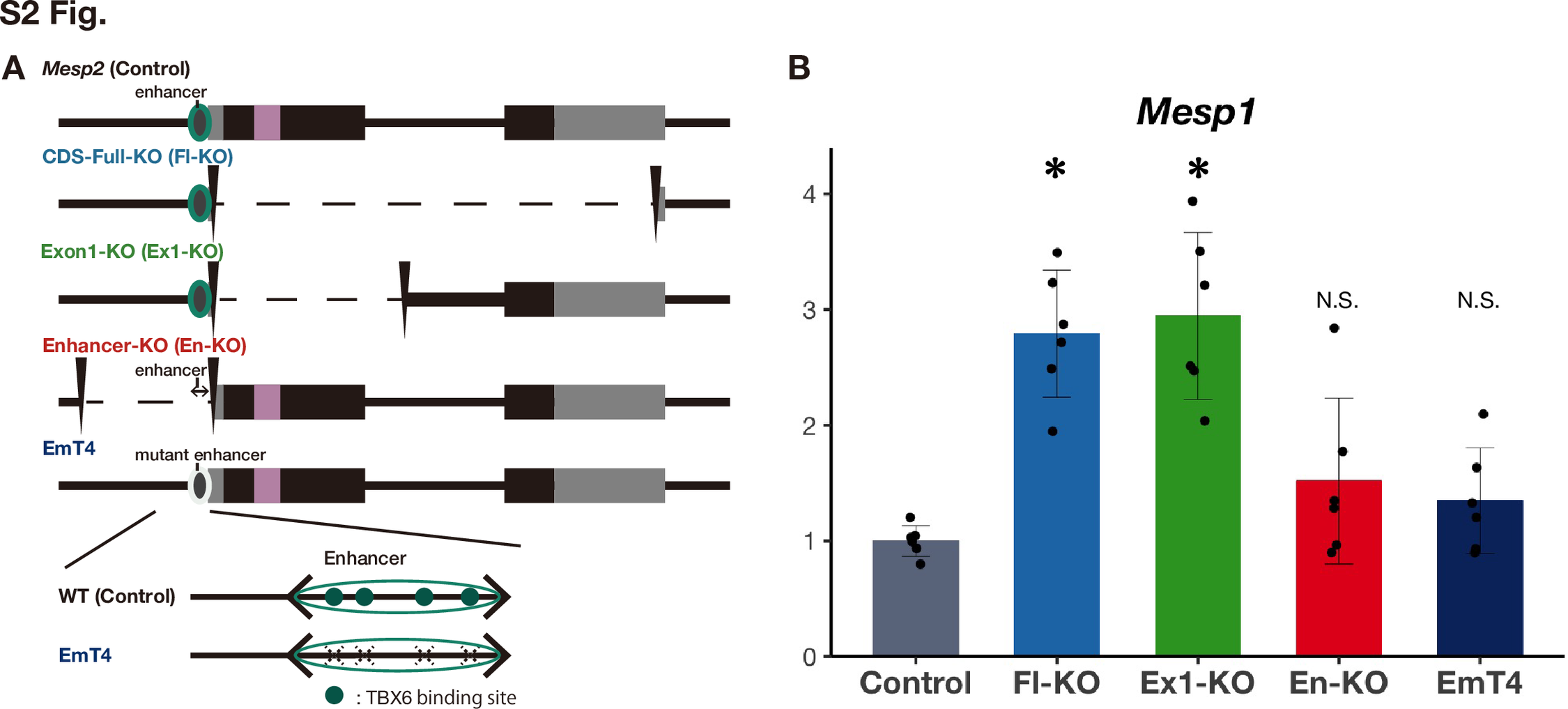

Supplement: S2 Fig — A Schematic diagram of Mesp2 gene structure and its deletions or mutations. B Summary of Mesp1 expression in Mesp2 mutant lines. The data of Mesp1 expression are derived from: control, CDS-Full-KO, and Exon1-KO from Fig 1G; P2-Enhancer-KO from Fig 2D; EmT4 from Fig 2G. These data were normalized by Mesp1 expression in the control in each experiment. Asterisk indicates significant (p < 0.05). (TIF) [file pgen.1010000.s002.tif]

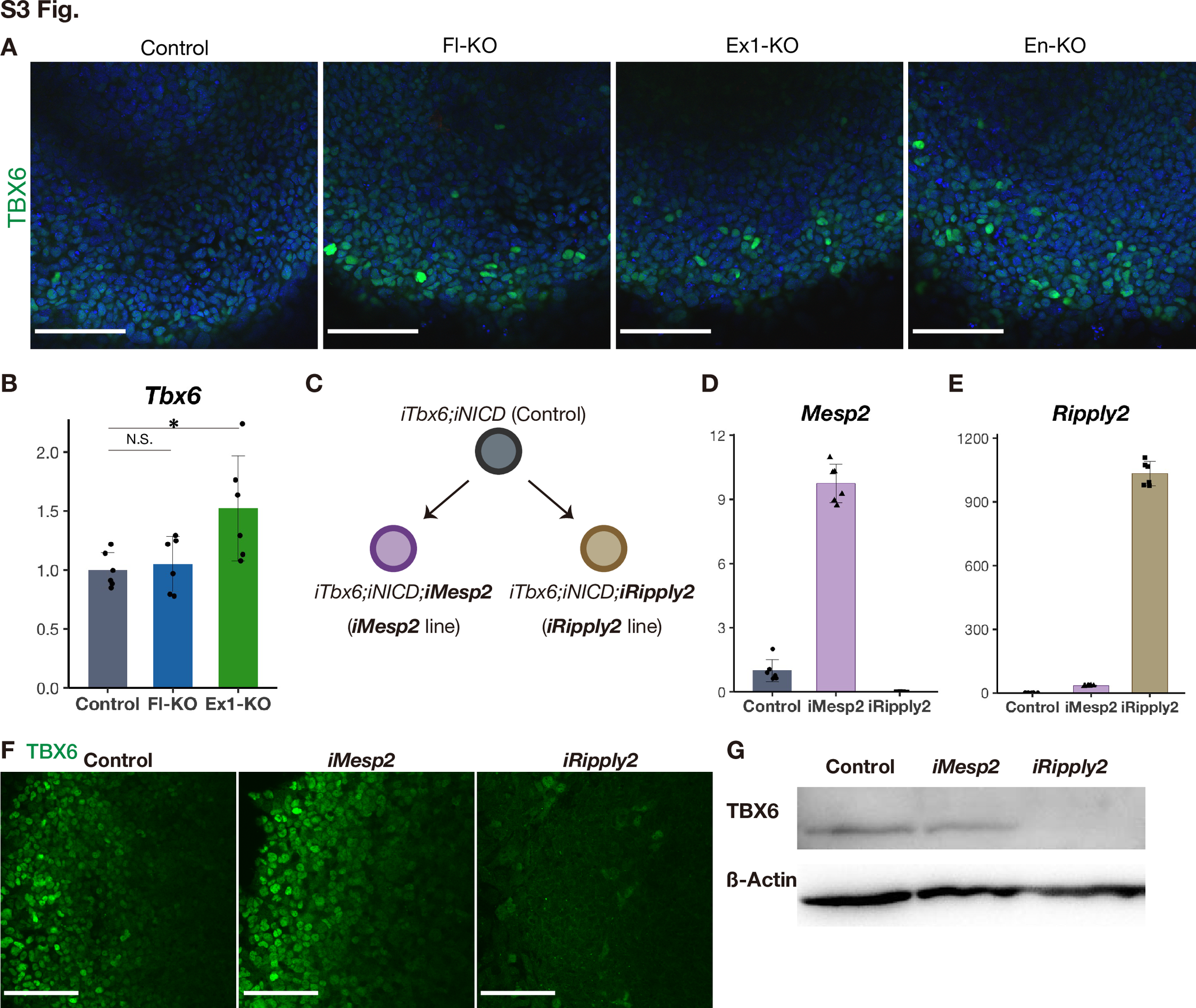

Supplement: S3 Fig — A Immunostaining of TBX6 in the in vitro PSM of iTbx6;iNICD (control), iTbx6;iNICD;Mesp2-CDS-KO, iTbx6;iNICD;Mesp2-Exon1-KO, and iTbx6;iNICD;Mesp2-Enhancer-KO upon Dox administration. Scale bars: 100 μm. B qPCR analysis of Tbx6 in the in vitro PSM (n = 6 cultures for each genotype). The expression level is presented as the ratio against iTbx6;iNICD line (control). P-values were calculated by the Mann-Whitney U test comparing iTbx6;iNICD (control) and iTbx6;iNICD;Mesp2-KO lines. Data are presented as the mean ± SD. Asterisk indicates significant (p < 0.05). C Schematic illustration of the generation of iMesp2 line and iRipply2 lines. D, E qPCR analysis of Mesp2 (D) and Ripply2 (E) in the in vitro PSM (n = 6 cultures for each genotype). The expression level is presented as the ratio against iTbx6;iNICD line (control). F Immunostaining of TBX6 in the in vitro PSM of iTbx6;iNICD (control) (left), iTbx6;iNICD;iMesp2 (middle), and iTbx6;iNICD;iRipply2 (right) upon Dox administration. Scale bars: 100 μm. G Western blotting analysis to monitor the degradation of TBX6 in the in vitro PSM of iTbx6;iNICD (control), iTbx6;iNICD;iMesp2, and iTbx6;iNICD;iRipply2 upon Dox administration. ß-Actin was used as the internal control of the protein lysate. (TIF) [file pgen.1010000.s003.tif]

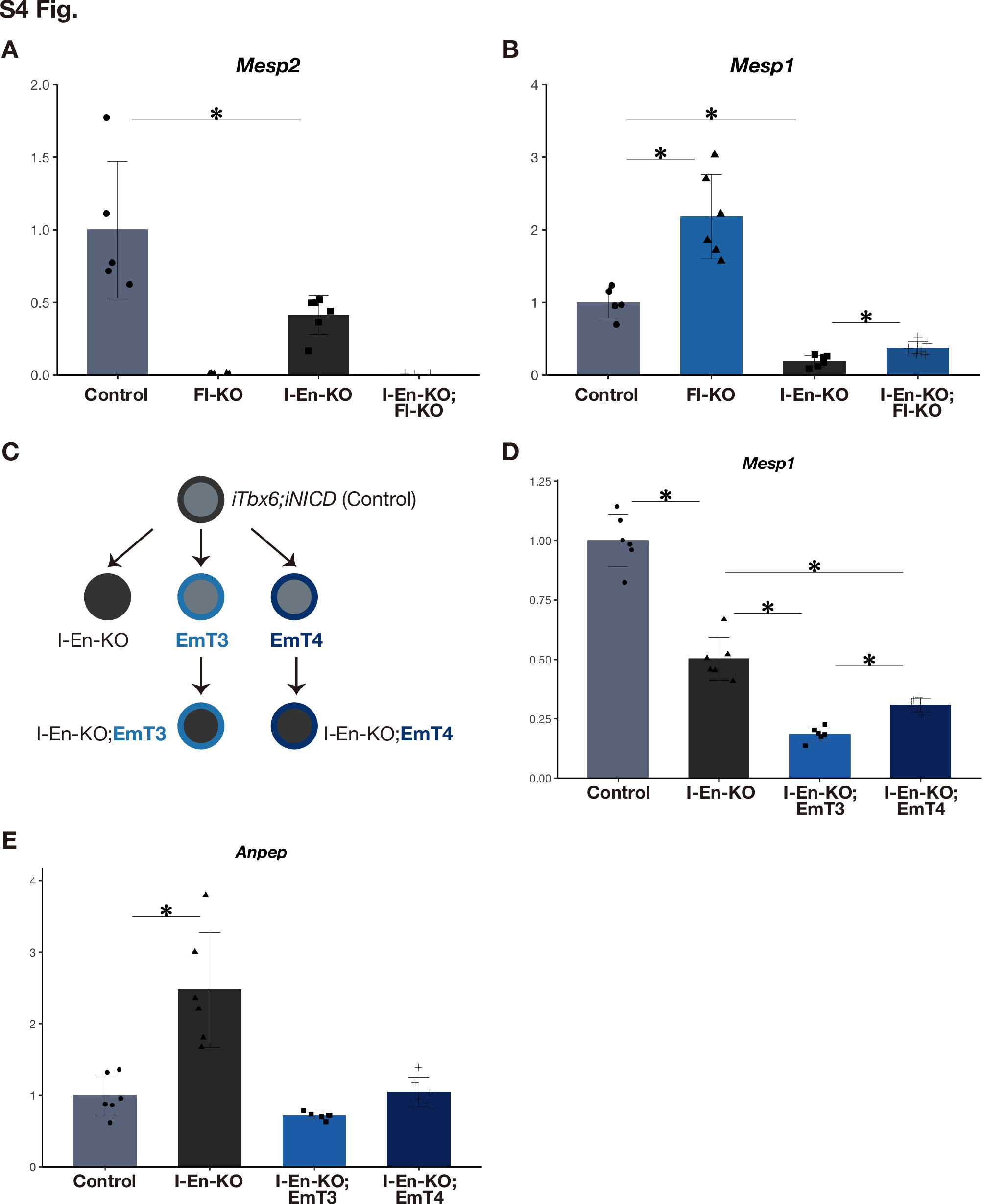

Supplement: S4 Fig — A, B qPCR analysis of Mesp2 (A) and Mesp1 (B) in in vitro PSM; iTbx6;iNICD (control), iTbx6;iNICD;I-enhancer-KO, Mesp2-CDS-Full-KO with the intact I-enhancer, and additive Mesp2-CDS-Full-KO on iTbx6;iNICD;I-enhancer-KO (n = 5 or 6 cultures for each genotype). The expression levels are presented as the ratio against iTbx6;iNICD line (control). P-values were calculated by the Mann-Whitney U test comparing iTbx6;iNICD;I-enhancer-KO and others. Data are presented as the mean ± SD. Asterisk indicates significant (p < 0.05). C Schematic illustration of the generation of I-enhancer-KO with additive P2-enhancer mutations (EmT3 or EmT4). D qPCR analysis of Mesp1 in the in vitro PSM; iTbx6;iNICD (control), iTbx6;iNICD;I-enhancer-KO, and iTbx6;iNICD;I-enhancer-KO with additive P2-enhancer mutations (EmT3 or EmT4) (n = 6 cultures for each genotype). The expression levels are presented as the ratio against iTbx6;iNICD line (control). P-values were calculated by the Mann-Whitney U test comparing samples indicated in this figure. Data are presented as the mean ± SD. Asterisk indicates significant (p < 0.05). (TIF) [file pgen.1010000.s004.tif]

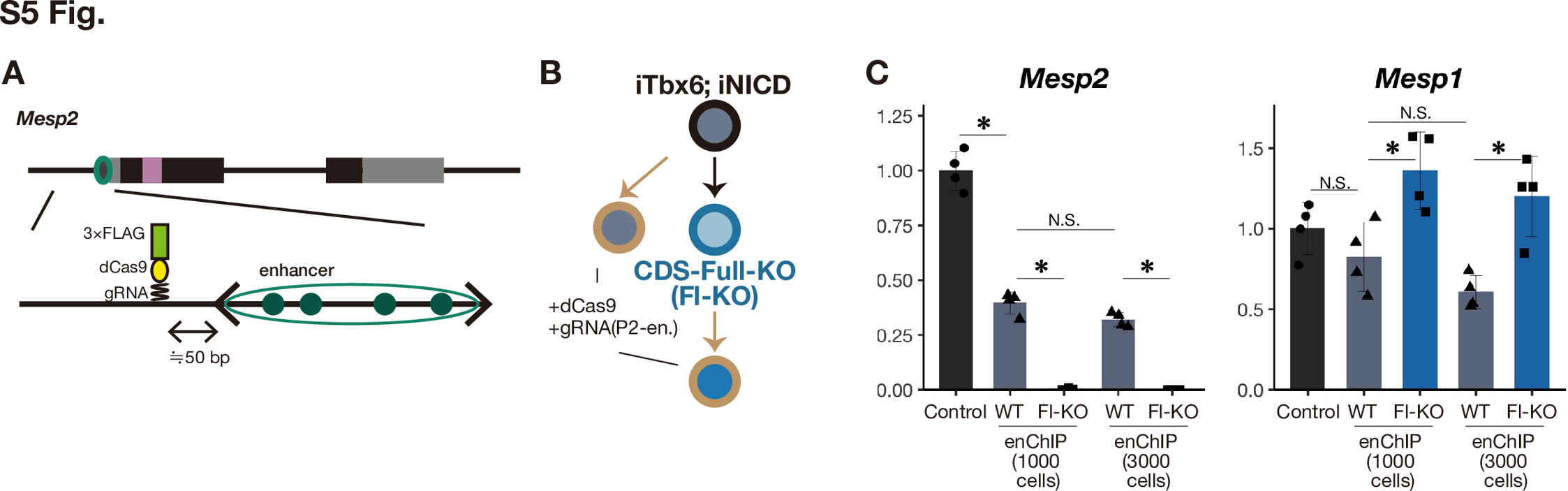

Supplement: S5 Fig — A Schematic diagram of the gRNA recognition position proximal to the P2-enhancer, which was used for enChIP lines. gRNA sequences are listed in S1 Table. B Schematic illustration of the generation of enChIP lines. C qPCR analysis of Mesp2 and Mesp1 in the in vitro PSM; iTbx6;iNICD (control), and enChIP lines (n = 4 cultures for each genotype). The starting cell number used for control PSM induction per well was 1000. For enChIP cell lines, 3000 cells were also used to induce PSM. The expression of Mesp genes in PSM from 1000 or 3000 cells was not different. The expression levels are presented as the ratio against iTbx6;iNICD line (control). P-values were calculated by the Mann-Whitney U test comparing samples indicated in this figure. Data are presented as the mean ± SD. Asterisk indicates significant (p < 0.05). (TIF) [file pgen.1010000.s005.tif]

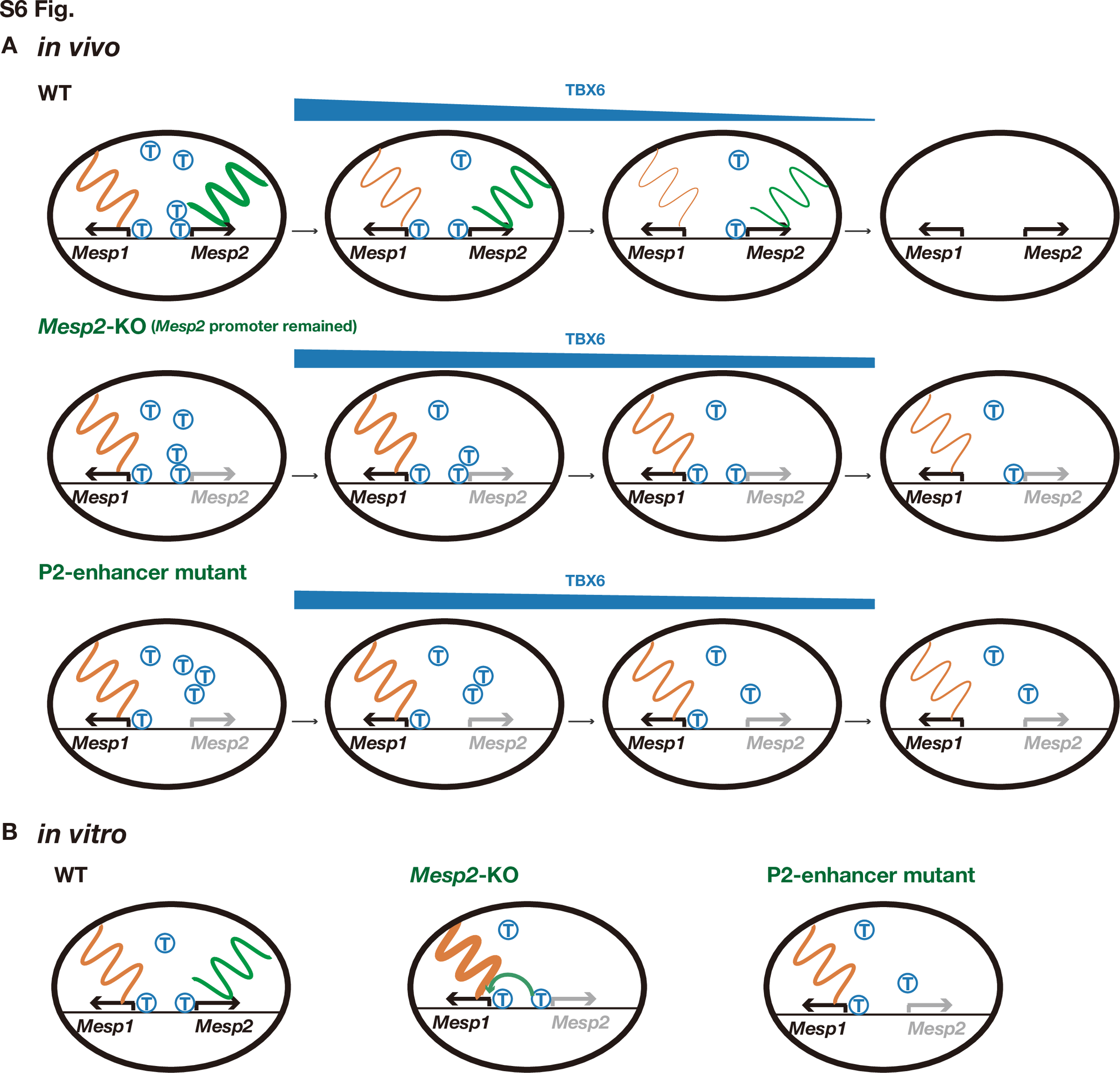

Supplement: S6 Fig — Schematic illustration of the course of Mesp1 and Mesp2 expression and TBX6 amount in in vivo (A) and in vitro (B) PSM. Transcripts of Mesp1 and Mesp2 are shown as orange and green wavy lines, respectively, and their thickness indicates the strength of transcription. TBX6 protein is shown as a blue T. Binding sites of T on the line indicate I- or P2-enhancer regions. Note that the position of the I-enhancer region is virtually described and not accurate. A Schematic illustration of cells undergoing somitogenesis in vivo. TBX6 is gradually degraded by the RIPPLY2-mediated proteasome pathway, and Mesp1 and Mesp2 are also gradually degraded in WT. On the other hand, TBX6 remains [18,20,22] and may continue to bind the I-enhancer in Mesp2-KO and P2-enhancer mutant mice. The duration of TBX6 binding to the Mesp1-enhancer is prolonged, which can increase the amount of Mesp1 transcripts. B Schematic illustration of cells after the TBX6 and NICD induction in vitro. TBX6 is not fully degraded even in the control due to the exogenous induction of TBX6. The TBX6 binding site on the I-enhancer may be occupied in the control and Mesp2-KO conditions. The I-enhancer may always be activated and not differ between the control and Mesp2-KO conditions. (TIF) [file pgen.1010000.s006.tif]
