# Supplementary material for: Repurposing of the enhancer-promoter communication underlies the compensation of Mesp2 by Mesp1
Source: PLoS Genet. 2022 Jan 13;18(1):e1010000. doi: 10.1371/journal.pgen.1010000 (PMC8791502; doi:10.1371/journal.pgen.1010000)
Supplement: Attachment — Submitted filename: Point_by_point_response.docx [file pgen.1010000.s011.docx]

**Point-by-Point Response to the Reviewers’ Comments**

TITLE: Repurposing of the enhancer-promoter communication underlies the compensation of *Mesp2* by *Mesp1*

H. Okada and Y. Saga

We greatly appreciate the editors and reviewers for their time and effort to handle and evaluate our manuscript. We were greatly encouraged by the overall positive opinions, and the comments from the referees were valuable and improved the quality of the manuscript.

In our revised manuscript and the point-by-point responses, we renamed the *Mesp1*-enhancer and the *Mesp2*-enhancer as I-enhancer and P2-enhancer, respectively.

Specific concerns raised by the reviewers were addressed individually as follows:

**Reviewer 1**

**Concerns:**

1. **“The negative feedback loop of TBX6-Mesp2-Ripply2 in the regulation of Mesp2 and Mesp1 expression suggests that total amount of Mesp1 and Mesp2 is very important for the presomitic mesoderm differentiation. This mechanism is not only for the compensation of loss-function of Mesp2, but also may happen when the expression of Mesp2 is too much. Therefore, it is necessary to determine whether the overexpression of Mesp2 would downregulate the expression of Mesp1 during PSM differentiation. If it is true, the findings would be more significant. Please the authors discuss this point.”**

   We appreciate the reviewer’s comments on this important issue. The negative feedback loop of TBX6-MESP2-RIPPLY2 may regulate the expression of not only *Mesp2,* but also *Mesp1* at least *in vivo* because the I-enhancer is also regulated by TBX6 (see figure provided below, panels A and B). In the previous manuscript, we showed that TBX6 protein expression was increased in the *Mesp2*-KO, and we explained that this is due to the breakdown of the negative feedback loop, which may increase *Mesp2*-enhancer activity, leading to a compensation response. However, we have to retract our original claim. To validate this more quantitatively, we reexamined the TBX6 protein level in *Mesp2*-Exon1-KO using Western blotting and immunocytochemistry, and found that TBX6 does not always increase in the *Mesp2*-Exon1-KO line compared with the control line (Figure below, panels C and D), which conflicts with our previous data shown in Fig 3C and S3A Fig of our original manuscript. Importantly, however, we confirmed that *Mesp1* is always upregulated in the *Mesp2*-Exon1-KO line, indicating a compensation response (Figure below, panel E). Thus, we concluded that the protein level of TBX6 is variable in the PSM induction system, but this does not affect the compensatory response. We think that the negative feedback loop of TBX6-MESP2-RIPPLY2 does not occur in our *in vitro* system because of the continuous artificial induction of TBX6 via the Dox system. Therefore, we withdrew the description of the relevance of the negative feedback loop to genetic compensation, and revised our manuscript and Fig 3.

2. **“Authors should provide more evidence for the negative feedback model in the figure 3. For instance, authors can investigate whether the overexpression of Ripply2 in Ex1-KO and En-KO cells would downregulate the level of TBX6 protein.”**

   We understand the reviewer’s interest in the negative feedback model in our *in vitro* system. However, as mentioned in the last section, we do not think that the negative feedback loop functions properly in our *in vitro* system. Nevertheless, it remains unclear whether the TBX6 protein was not degraded due to an insufficient amount of RIPPLY2. Thus, we followed the reviewer’s suggestion and established inducible *Mesp2* and *Ripply2* expression lines by manipulating *iTbx6;iNICD* lines, referred to as *iMesp2 and* *iRipply2* lines, using the PiggyBac transposon system (see also figure provided below, panel A). As expected, *Mesp2* and *Ripply2* were strongly induced in each line (Figure below, panels B and C). Next, we examined the level of TBX6 protein using these lines. As expected, TBX6 was almost completely suppressed in the *iRipply2* line, confirmed by Western blotting and immunocytochemistry analyses (Figure below, panels D and E). However, the degradation of TBX6 protein was not accelerated in the *iMesp2* line compared with the control line (*iTbx6;iNICD*) (Figure below, panels D and E), even though a greater than thirty-fold increase in the amount of *Ripply2* (still low compared with the *iRipply2* line) was induced by the overexpression of *Mesp2* (Figure below, panels C’), indicating that the negative feedback regulation of TBX6 was not induced even in the *iMesp2* line. In summary, we concluded that RIPPLY2 can suppress TBX6 at the protein level, but the amount of induced RIPPLY2 in the control and *iMesp2* lines was insufficient to degrade TBX6 in our system. We added this data in S3 Fig.
   We hope that the reviewer agrees with our conclusion.

3. **“Using en-ChIP method, authors demonstrated that Mesp2-enhancer physically interacts with Mesp1 and Anpep promoters and Mesp1-enhancer in the PSM but not in ES cells, which is independent of TBX6. The results suggest that topologically associating domains (TADs) have changed from ES cells to the PSM. Authors may find evidence from the 3D map in published data base.”**

   We agree with your speculation because TAD formation is established along with mammalian development [1]. Such TAD formation may aid in the establishment of E-P communications inside TAD. However, we were unable to find a 3D map of the PSM. This speculation will be challenged in a future study.

**Reviewer 2**

**Concerns:**

1. **“the enChIP-based results are highly variable and not well controlled as the modest increases in contact frequency could be explained simply by the local chromatin environment in the PSM and not necessarily increased contacts. Most importantly the weak dynamic range of the assay cannot convincingly exclude a change in interaction frequency if this were at play. Indeed no manipulation changes the measured (inferred) interaction frequency. The interaction data should be removed.”**

   We appreciate this critical comment. As this reviewer pointed out, the range in our enChIP assay was weak (See figure below, panel A and B). Thus, we reevaluated the authenticity of our experiment.
   First, we compared the interaction of the P2-enhancer with the I-enhancer (*Mesp1*-enhancer in original submission) and NC_11.5k, both of which are located at 11.5k bp from the P2-enhancer on opposite sides. Although the interaction with I-enhancer was significantly higher than that with NC_11.5k in the *Mesp2*-KO PSM, this was not the case in the control PSM (Figure below, panel C); therefore, we cannot claim that the I-enhancer specifically interacted with the P2-enhancer more in the PSM. However, the interaction with I-enhancer was higher in the PSMs than in ES cells, as stated by the reviewer, suggesting that the local chromatin condition in the PSM affects the interaction of the P2-enhancer with the surrounding environment. We agree with the reviewer’s comment on the interaction with the I-enhancer.
   However, we additionally examined the interaction with NC_17k, which is the same distance to the *Mesp1* promoter and more distant from P2-enhancer than NC_11.5k (Figure below, panel A). The interaction of P2-enhancer with NC_17k was lower than in the case of the NC_11.5k and was comparable with that with NC_60M in the PSM (Figure below, panel D), indicating that we were detecting a meaningful interaction between the *Mesp1*-promoter and P2 enhancer. Thus, we modified our claim and revised our manuscript by focusing on the interaction of the P2-enhancer with the *Mesp1* promoter rather than the I-enhancer (previous *Mesp1*-enhancer). We think that these data are essential to support our hypothesis. We hope that this revision is acceptable for the reviewer.

2. **“The model is confusing as it is inconsistent between Fig 5A and 6A.”**

We apologize for confusing the reviewer. We just presented Fig 5A as a working model of the physical interaction assay, which was negated, and presented Fig 6A as the final proposal model. We added a description of the “working model” in Fig 5A and 5B. The description of line 276 in the original version was also likely confusing; therefore, we also revised this description, too. We hope that these revisions will make sense to the readers.

**Reviewer 3**

**Major concerns:**

1. **“Introduction: There are several pieces of information that would be nice to include in the introduction. I am not an expert in PSM biology, and I had a hard time understanding what the normal expression pattern of Mesp1 is and the phrase starting on line 98. Specifically, the “reciprocal transcription termination of Mesp2” and the “temporal activation and termination of the Mesp2 gene”. Does termination mean repression? If so, I think repression is a better word, since it implies transcription of Mesp2 is blocked, not initiated and then terminated. In addition, the diagram in Figure 3A was helpful – can this or a variant of this be introduced sooner?”**

   We appreciate the reviewer for pointing out the lack and uncertainty in our descriptions and apologize for the difficulty in understanding. Following these suggestions, we revised our manuscript by adding that *Mesp1* is simultaneously expressed with *Mesp2* in the same region in the PSM, correcting the words “termination” in line 98 in the original manuscript to “repression,” and adding the schematic diagram of the negative feedback loop of TBX6-MESP2-RIPPLY2 in Fig 1B. (As a consequence, the following panels in the original manuscript were renumbered.) We hope that these changes satisfy the reviewer.
2. **“Identification of enhancers and terminology used to refer to them: At the heart of this paper is the claim that the Mesp2-enhancer can up-regulate Mesp1 in the Mesp2 KO condition. Therefore, much more detail should be provided in to how the Mesp1- and Mesp2-enhancers were identified and what evidence was used to assign them to Mesp1 and Mesp2. Was it simply proximity? Were the enhancers cloned into reporters to determine their expression patterns? In the description, limitations to the previous assignment of enhancers to genes should be discussed.”**

   We agree that we must provide information about the *Mesp*-enhancers. The enhancers were originally identified by a reporter assay (see Figure below, two examples, a and b in panel A). To explore the necessity of these elements in a more reliable genetic context, a bacterial artificial chromosome transgene (BAC TG) method was used. The BAC TG contains the 160k bp spanning the genomic context, including reporter genes in the *Mesp1* or *Mesp2* coding regions (Figure below, an example in panel B). Thus, these regions were assigned to the regulatory region of each *Mesp* gene. However, this methodological approach was unable to provide evidence that redundant enhancers regulate the other *Mesp* gene. Moreover, *Mesp* genes are almost simultaneously expressed in the same regions (Figure below, panel C from M. Morimoto *et al., Dev. Biol.*, 2006 [2]), which makes it difficult to distinguish the enhancer activities for each *Mesp* gene by the expression patterns.
   We added the description of the information mentioned here in the manuscript.

3. **“Reference to previous work: There are two related but distinct concepts that this work touches upon. The first is shadow enhancers –redundant enhancers that control a single gene in the same cell type. This term was coined to describe some Drosophila enhancers (https://pubmed.ncbi.nlm.nih.gov/18772429/), but has also been widely discussed in reference to mammalian genes (e.g. https://pubmed.ncbi.nlm.nih.gov/27918583/, reviewed here: https://pubmed.ncbi.nlm.nih.gov/33442000/ and here: https://pubmed.ncbi.nlm.nih.gov/22083793/). This term seems, at a minimum, to apply to the Mesp1 and Mesp2 enhancers in relation to the regulation of Mesp1, but given the data in Figure 4, also to the regulation of Mesp2. The second is the presence of enhancers that are looped to and activate multiple genes. This was described here: https://www.nature.com/articles/nature13417, among other references. This work should both reference and discuss the relationship of the present work to these previous papers.”**

   We appreciate these critical suggestions. We agree that these previous works are closely relevant to our work, and must be cited and discussed. Regarding the first one, shadow enhancers, the I- and P2-enhancers resemble shadow enhancers for both *Mesp* genes. We acquired new data following suggestion #4 by this reviewer that the P2-enhancer functions in *Mesp1* regulation in the I-enhancer-KO condition, suggesting that I-enhancer deletion was the trigger for the *Mesp2*-enhancer to act on the *Mesp1* promoter. As the I- and the P2-enhancer synergistically upregulate *Mesp1* expression for genetic compensation, these shadow enhancers may function in several ways. We discussed this point. Regarding the second comment, how an enhancer activates multiple target genes that interact with the enhancer, this is a fundamental question about the mechanism of genetic compensation. Thus, we cited recent papers and discussed this point. We added a section, “**The mode of the *Mesp*-enhancers as shadow enhancers,**” in the discussion section, and we also discussed the second issue in **”The enhancer competition theory is one explanation for the repurposing of enhancer-promoter communication.**” We hope this revision satisfies the reviewer.
4. **“More careful description of the necessity/sufficiency of enhancers: Given the results seen with shadow enhancers, I would suggest being a bit more guarded in the interpretation that the Mesp2-enhancer does not regulate Mesp1 under physiological conditions (line 212, line 381). I think it would be more accurate to say that the Mesp2-enhancer is not necessary under physiological conditions to drive WT levels of Mesp1, but since only a single cell type/time point was assayed, it is possible that the Mesp2-enhancer does regulate Mesp1, even in control conditions, or that it contributes to dynamic aspects of its regulation. Related to this issue, do you think that the non-zero expression of Mesp1 in the P1E-KO is due to the action of the Mesp2-enhancer serving as a backup/shadow enhancer?”**

   We greatly appreciate this reviewer’s critical suggestion for the interpretation of P2-enhancer activity dynamics, and we agree that the enhancer’s dynamics are also related to its function as a shadow enhancer. To determine whether the P2-enhancer activates the *Mesp1* expression in the condition where the primary enhancer for *Mesp1* is disrupted, we added the inactivated P2-enhancer to the I-enhancer deletion and examined the *Mesp1* expression (see Figure below, panel A). Of note, *Mesp1* was downregulated, which indicates that the P2-enhancer also acts on *Mesp1* in the absence of its own enhancer. We added this data in S4C and S4D Fig.
   However, *Mesp1* expression was not completely suppressed by this combinatorial inactivation of the I- and P2- enhancers, suggesting unknown regulatory elements in *Mesp1* expression. A study reporting the transcriptional role of T and Eomes, which are T-box family proteins, showed the DNA elements on which T and Eomes bind, corresponding to the I- and P2-enhancers (Figure below, orange and green rectangle regions, respectively, in panel C from Tosic *et al.*, *Nat. Cell Biol.*, 2019 [3]). This study also suggested the potential enhancer elements (Figure below, magenta rectangle regions in panel C) by presenting the evidence of binding activity of these transcription factors, their open chromatin accessibility, and accumulation of an enhancer histone marker H3K27ac. Thus, these regions can also function as enhancers and play a role in *Mesp* genes regulation.
   Thanks to the reviewer’s suggestion, we revealed another aspect of the dynamics of the P2-enhancer. We greatly appreciate this excellent comment and hope our answer is satisfactory.

5. **“Description of qPCR results/methodology: Compared to standards like the MIQE ones, the description of the analysis of the qPCR results seems a bit lacking in detail. The units should be specified on all y-axes that show qPCR results.
   I also think it would make the graphs more interpretable if the y-axis had the same range in all qPCR figures (or at least in panels in one figure).** **Since results were normalized to Gapdh, it should also be possible to compare the expression of Mesp1 and Mesp2, which may be of interest.”**

   We agree that the description about the qPCR results/methodology was lacking. According to these suggestions and MIQE standards, we added the following information to the Materials and Methods part: the PCR condition, the calculation method of qPCR fluorescence data, the PCR efficiency of each PCR primer set, and statement of primer specificity was confirmed by the single melting peaks of PCR amplicons.
   Although we evaluated the high PCR efficiency within the range of our qPCR assay, we performed the qPCR analysis using the 2^-ΔΔCq^ method without standard curves because we analyzed multiple samples in a duplicate set at the same time. For this reason, we avoid comparing other PCR results from different run sets, for example, Fig 2C and 2D. However, to make the graphs more interpretable, we summarized the qPCR data normalized by control data, in S2 Fig. Moreover, we did not quantify the absolute amount of cDNA, which prevented us from comparing other genes, such as *Mesp1* and *Mesp2,* because the PCR efficiency of each gene primer set is not equal. Thus, we showed the relative gene expression levels normalized by Gapdh expression and the ratio to the control samples for each gene in each experimental set.

**Minor comments:**

1. **I might choose to call Mesp1 and Mesp2 paralogs instead of homologs, since the term is more specific.**

   We agree with this suggestion for the specific definition of *Mesp* genes because these genes originated from gene duplication and different genes. We changed the word “homologs” to “paralogs” according to this suggestion.
2. **Line 58 – “interacted” should be “interacting”**

   We appreciate the reviewer’s comment and fixed this grammatical error.
3. **Line 75 – the claim is made that NMD is a widely used mechanism of genetic compensation, but no evidence is cited. Has someone actually tried to systematically observe this? Or is this based on the presence of multiple examples? Please clarify.**

   We apologize for the confusion. There are two individual studies that demonstrated that NMD is required for genetic compensation of several genes. Some genes require NMD, but the studies never mentioned that NMD always induces genetic compensation, which is unlikely. In summary, this NMD-mediated model is supported by the presence of multiple examples, but not systematically. We revised the manuscript for clarification and cited these papers.
4. **Line 80 – I might call this replacement of the Mesp2 coding region, instead of locus, since locus implies regulatory information was also included (unless it was?)**

   We appreciate the reviewer’s careful read of our manuscript and suggestion of a precise description. We agree with this suggestion and corrected “*Mesp2* coding locus” to “*Mesp2* coding region.”
5. **Line 92 – should “domains” actually be “motifs”?**

   Thank you for noting our word choice. “Domain” indicates a specific protein module, but the specific DNA sequence, such as sites transcription factors bind, is termed “motif.” We revised our manuscript accordingly.
6. **Line 113 – To make this more accessible to a broad audience, I might briefly include a description of the published PSM induction system. Otherwise, it is hard to understand the following sentence starting on line 114.**

   We appreciate the reviewer’s kind suggestion. Following this suggestion, we added a brief description about this *in vitro* PSM system. We hope that this description helps the readers understand our manuscript.
7. **Line 118 – Please explain the relationship between the Notch1 and NICD genes.**

   We added an explanation of this relationship in the manuscript.
8. **Line 167 – How were the binding sites disrupted? Is there a reason that Mesp1 is more highly expressed in the EmT4 than the EmT3 line?**

   We apologize for the lack of description on disrupting TBX6 binding sites. For the introduction of these mutations, we followed the method previously reported. Briefly, we prepared the P2-enhancer sequence with point mutations by previously reported nucleotide substitutions where TBX6 no longer binds in the targeting vector and transfected this as a recombination template with Cas9 and gRNA, which recognizes the TBX6 binding site in the P2-enhancer. Then, we selected the EmT3 and EmT4 lines. We added this explanation in the main text.
   We do not have a clear answer to the second question, “Is there a reason that *Mesp1* is more highly expressed in the EmT4 than the EmT3 line?”. However, in perspective of the shadow enhancer, other enhancer elements suggested by the previous study by Tosic *et al* [3]*.* (see also in Major concerns 4.) may activate *Mesp1* expression more in the *Mesp* depletion condition because *Mesp2* was more markedly suppressed in the EmT4 line than in the EmT3 line (See Fig 2F). In addition to the examination of other *Mesp1* regulatory elements, an integrative analysis of these enhancers is required in future studies.
9. **Line 190 – Is there a quantification of TBX6? Representative images seem a bit weak as evidence for the protein level differences.**

   We agree with this opinion. Thus, we added the quantification of TBX6 by Western blotting (WB) analysis. As mentioned in the response to comment #1 by reviewer 1, we performed WB analysis in addition to the immunocytochemistry. However, the amount of TBX6 was not higher in the *Mesp2*-KO line (see Figures in Reviewer#1-1 section, panels C and D), conflicting with our first submitted data. However, *Mesp1* was upregulated in compensation (see Figure in Reviewer#1-1 section, panel E). Therefore, we did not change our claim, but we revised our manuscript as follows: this genetic compensation between *Mesp* genes is independent of the breakdown of the negative feedback loop.
10. **Line 191 – It may be useful to remind the reader of Ripply2’s mechanism of action to explain why protein and mRNA levels of Tbx6 are uncoupled.**

    We appreciate this kind suggestion. We added a reminder about the protein degradation function of RIPPLY2 upon TBX6 expression.
11. **Line 221 – is Anpep normally expressed in the PSM?**

    It has not been reported. Thus we examined the expression using reported single-cell RNA-seq data from A. Scialdone *et al., Nature,* 2016 [4]. *Anpep* is expressed in the PSM cluster that expresses *Mesp1*, *Mesp2,* and Tbx6. Thus, *Anpep* is expressed in the PSM.

12. **Line 231 – “replaced” should be “replacing”**

    We fixed this grammatical error as you suggested.
13. **Line 316 – I’m not sure “inherent” is the right word to use here**

    We agree with this opinion because this expression, “inherent gene,” is not used. We wanted to describe the unexpected genetic failure, although this is a redundant expression. We removed “inherent” from the text. We hope that this makes sense to the readers.
14. **Discussion of the regulation of E-P communication: There is more recent work from the Levine lab showing that some E-P distances can be large, even when actively transcribing:** [**https://www.pnas.org/content/116/30/15062**](https://www.pnas.org/content/116/30/15062)**. It might be nice to include this citation, though I do agree with the conclusion of this section in that we don’t really know how enhancers chose target promoters.**

    We appreciate this information as we missed this work. We cited this study and a related paper [5], and discussed how an enhancer selects its target among genes with E-P communications.

1.	Ke Y, Xu Y, Chen X, Feng S, Liu Z, Sun Y, et al. 3D Chromatin Structures of Mature Gametes and Structural Reprogramming during Mammalian Embryogenesis. Cell. 2017;170: 367-381.e20. doi:10.1016/j.cell.2017.06.029

2.	Morimoto M, Kiso M, Sasaki N, Saga Y. Cooperative Mesp activity is required for normal somitogenesis along the anterior-posterior axis. Dev Biol. 2006;300: 687–698. doi:10.1016/j.ydbio.2006.08.043

3.	Tosic J, Kim G-J, Pavlovic M, Schröder CM, Mersiowsky S-L, Barg M, et al. Eomes and Brachyury control pluripotency exit and germ-layer segregation by changing the chromatin state. Nat Cell Biol. 2019;21: 1518–1531. doi:10.1038/s41556-019-0423-1

4.	Scialdone A, Tanaka Y, Jawaid W, Moignard V, Wilson NK, Macaulay IC, et al. Resolving early mesoderm diversification through single-cell expression profiling. Nature. 2016;535: 289–293. doi:10.1038/nature18633

5.	Alexander JM, Guan J, Li B, Maliskova L, Song M, Shen Y, et al. Live-cell imaging reveals enhancer-dependent sox2 transcription in the absence of enhancer proximity. Elife. 2019;8: 1–42. doi:10.7554/eLife.41769
