# Supplementary material for: Repurposing of the enhancer-promoter communication underlies the compensation of Mesp2 by Mesp1
Source: PLoS Genet. 2022 Jan 13;18(1):e1010000. doi: 10.1371/journal.pgen.1010000 (PMC8791502; doi:10.1371/journal.pgen.1010000)
Supplement: Attachment — Submitted filename: Point_by_point_response_2nd.docx [file pgen.1010000.s012.docx]

**Point-by-Point Response to the Reviewers’ Comments**

TITLE: Repurposing of the enhancer-promoter communication underlies the compensation of *Mesp2* by *Mesp1*

H. Okada and Y. Saga

We greatly appreciate the editors and reviewers for their time and effort to handle and evaluate our manuscript.

Specific concerns raised by the reviewers were addressed individually as follows:

**Reviewer 1**

**Concerns:**

1. **In addition, a partial deletion in either P1- or P2-enhancer leads to reduction of Mesp1 expression. Thus, it raises a key question for this research, which is whether the compensation response is due to the deletion (or partial deletion) of Mesp2 gene (there is a possibility that this region contains a repressive element), or due to the loss-function of Mesp2 protein. To address this question, the authors might overexpress Mesp2 in the Mesp2-deletion cell lines or knockdown Mesp2 in the control cell line.**
2. **If the answer is due to the loss-function of Mesp2 protein, what is the proposed model for loss-function of Mesp2 to upregulate the expression of Mesp1.”**

   We appreciate the reviewer’s comments on this issue that the compensation could be due to the deletion of *Meps2* gene locus or the loss of the MESP2 protein function. We addressed this question using another *Mesp2* mutant line, where the *Mesp2* coding sequence was replaced by mVenus fluorescent protein gene, called mVenus line (see figure provided below, panel A). Using mVenus line, we induced the PSM differentiation and found that the *Mesp1* expression was not upregulated (Figure below, panel B). This negated the possibilities that the *Mesp2* gene sequence represses *Mesp1* expression and that the loss of the MESP2 protein function induces the compensation. Combined with the transcriptional repression from the *Mesp2* locus in the *Mesp2*-Exon1-KO line where genetic compensation occurs (Fig 2A), the transcriptional repression may trigger the alteration of the P2-enhancer targets from *Mesp2* to *Mesp1*.
   We hope that this result satisfies the reviewer.

**Reviewer 3**

**Concerns:**

1. **on Line 263 "Anpep expression varied but was not reduced" -- I'd simplify this to "Anpep expression was not significantly reduced"**

   We have revised it accordingly.
2. **I believe there is a mistake in the x-axis labels of Figure 4F-H -- the last bar is I-En-KO; Ex1-KO, right? P2 wasn't knocked out in these cells, was it?”**

   We appreciate your comment. As you mentioned, we did not knock out the P2-enhancer. Thus, we revised these labels from I-En-KO; P2-Ex1-KO to I-En-KO; Ex1-KO in Figure 4F-H.
